# Estrogen Signalling and the Metabolic Syndrome: Targeting the Hepatic Estrogen Receptor Alpha Action

**DOI:** 10.1371/journal.pone.0057458

**Published:** 2013-02-25

**Authors:** Marko Matic, Galyna Bryzgalova, Hui Gao, Per Antonson, Patricia Humire, Yoko Omoto, Neil Portwood, Camilla Pramfalk, Suad Efendic, Per-Olof Berggren, Jan-Åke Gustafsson, Karin Dahlman-Wright

**Affiliations:** 1 Department of Biosciences and Nutrition, Karolinska Institutet, Huddinge, Sweden; 2 The Rolf Luft Research Center for Diabetes and Endocrinology, Karolinska Institutet, Karolinska University Hospital L1, Stockholm, Sweden; 3 Center for Nuclear Receptors and Cell Signalling, University of Houston, Houston, Texas, United States of America; 4 Department of Laboratory Medicine, Karolinska Institutet, Stockholm, Sweden; University of Padova, Italy

## Abstract

An increasing body of evidence now links estrogenic signalling with the metabolic syndrome (MS). Despite the beneficial estrogenic effects in reversing some of the MS symptoms, the underlying mechanisms remain largely undiscovered. We have previously shown that total estrogen receptor alpha (ERα) knockout (KO) mice exhibit hepatic insulin resistance. To determine whether liver-selective ablation of ERα recapitulates metabolic phenotypes of ERKO mice we generated a liver-selective ERαKO mouse model, LERKO. We demonstrate that LERKO mice have efficient reduction of ERα selectively within the liver. However, LERKO and wild type control mice do not differ in body weight, and have a comparable hormone profile as well as insulin and glucose response, even when challenged with a high fat diet. Furthermore, LERKO mice display very minor changes in their hepatic transcript profile. Collectively, our findings indicate that hepatic ERα action may not be the responsible factor for the previously identified hepatic insulin resistance in ERαKO mice.

## Introduction

The metabolic syndrome (MS) refers to a group of interrelated metabolic abnormalities including insulin resistance, increased body weight and abdominal fat accumulation, mild dyslipidemia and hypertension [1], [2], [3], [4]. Individuals with MS are at increased risk of cardiovascular disease and Type 2 diabetes (T2D) [5]. MS prevalence is on the rise worldwide, and has been correlated with an increased incidence of obesity resulting from a combination of a sedentary lifestyle and high energy diets [2]. Since a unifying mechanism underpinning the complex pathways leading to MS abnormalities remains undiscovered, present treatment regimes target MS symptoms through therapeutic intervention and lifestyle changes. Thus a better understanding of the underlying MS mechanisms is likely to provide a basis for development of more effective therapeutic strategies in MS treatment.

A growing body of evidence now demonstrates that estrogenic signalling can have an important role in MS development. Studies in both humans and rodents suggest that altered levels of estrogen or its receptors can lead to MS symptoms. For example, postmenopausal women, experiencing naturally decreased estrogen levels, are three times more likely to develop MS abnormalities than premenopausal women [6]. Furthermore, estrogen/progestin based hormone replacement therapy in postmenopausal women has been shown to lower visceral adipose tissue, fasting serum glucose and insulin levels [7]. Clinical observations in an estrogen receptor alpha (ERα) deficient male noted the development of hyperinsulinemia, impaired glucose tolerance (IGT), insulin resistance (IR) and increased body weight [8]. Additional cases show that men with decreased levels of aromatase, the principal enzyme of estrogen production, develop abdominal obesity, elevated blood lipids and IR, reviewed in [9], [10]. Rodent studies demonstrate that whole body ERα knockout (KO) (ERαKO) models have increased body weight, IGT, and IR [11], [12]. Aromatase KO mice diplay IR, IGT and increased abdominal fat, which are reversible by 17β-estradiol (E2) treatment [13], [14]. Ovariectomy, resulting in low estrogen levels, leads to increased body weight, increased basal blood glucose and IGT which are reversible by reintroduction of estrogen [15], [16], [17], [18]. The beneficial effect of estrogen in relation to normalising body weight and glucose homeostasis is further evidenced in ob/ob and high fat diet (HFD) fed mice, models of obesity and T2D. In both models, estrogen treatment improves glucose tolerance and insulin sensitivity [4], [19], [20], in addition to having a weight lowering effect in HFD-fed mice [20]. Collectively, these studies firmly establish a role for estrogenic signalling in the development of MS. However, these observations are derived from models with altered estrogenic action throughout multiple organs/tissues. This makes it difficult to correlate the sequences of events and tissue-specific contributions of the underlying estrogenic mechanisms to the observed phenotypes.

Estrogen signalling can be mediated by multiple receptors. Most of the known estrogenic effects are mediated via direct interaction of estrogen with the DNA-binding transcription factors, ERα and estrogen receptor beta (ERβ) [21], [22]. The resulting mechanism supports a ligand-modulated, ER-mediated, transcriptional gene regulation. Studies of whole-body ERβKO mice have shown that they do not exhibit altered insulin sensitivity and/or alterations in body weight [12]. However, some evidence exists that ERβ might still contribute to the development of MS in older mice and/or under specific metabolic conditions [23]. In contrast, ERα-selective signalling has clearly been associated with the MS. In addition to the observations from ERαKO mice [11], [12], selective ablations of ERα in the hypothalamic brain region or the hematopoietic/myeloid cells have both been reported to give rise to an increase in body weight and attenuated glucose tolerance [24], [25], [26]. Furthermore, treatment of ob/ob mice with the ERα-selective agonist propyl pyrazole triol (PPT) improved glucose tolerance and insulin sensitivity, supporting the importance of ERα action in the control of glucose and insulin function. In addition, estrogenic signalling has also been shown to occur via a membrane-bound G protein-coupled receptor (GPR) 30 [27] which has been implicated as an important factor in insulin production and glucose homeostasis [28].

Previously, using the euglycaemic–hyperinsulinaemic clamp, we showed that ERαKO mice exhibit defective insulin-mediated suppression of endogenous glucose production (EGP)[12]. Since the liver is the principal organ of EGP [29], [30], [31], we proposed that hepatic IR contributes to the observed IGT and IR in ERαKO mice. To further investigate the role of hepatic estrogenic action in the maintenance of hepatic glucose homeostasis, we now report the generation and characterisation of a liver-selective ERαKO mouse model, LERKO. We demonstrate that LERKO mice display efficient down-regulation of ERα expression selectively within the liver. LERKO body weight, hormone profiles as well as the glucose and insulin response are comparable to those of control (CT) animals even when challenged with HFD and/or aging. In addition comparative analysis of the hepatic transcriptional profile in LERKO animals with that of ERαKO animals showed that LERKO mice do not exhibit the changes observed in ERαKO mice. We henceforth speculate that the previously observed ERα-mediated hepatic insulin resistance in ERαKO mice occurs as a secondary effect in the development of MS abnormalities.

## Results

### The LERKO mouse model demonstrates liver-selective ERα ablation

**Figure 1:**
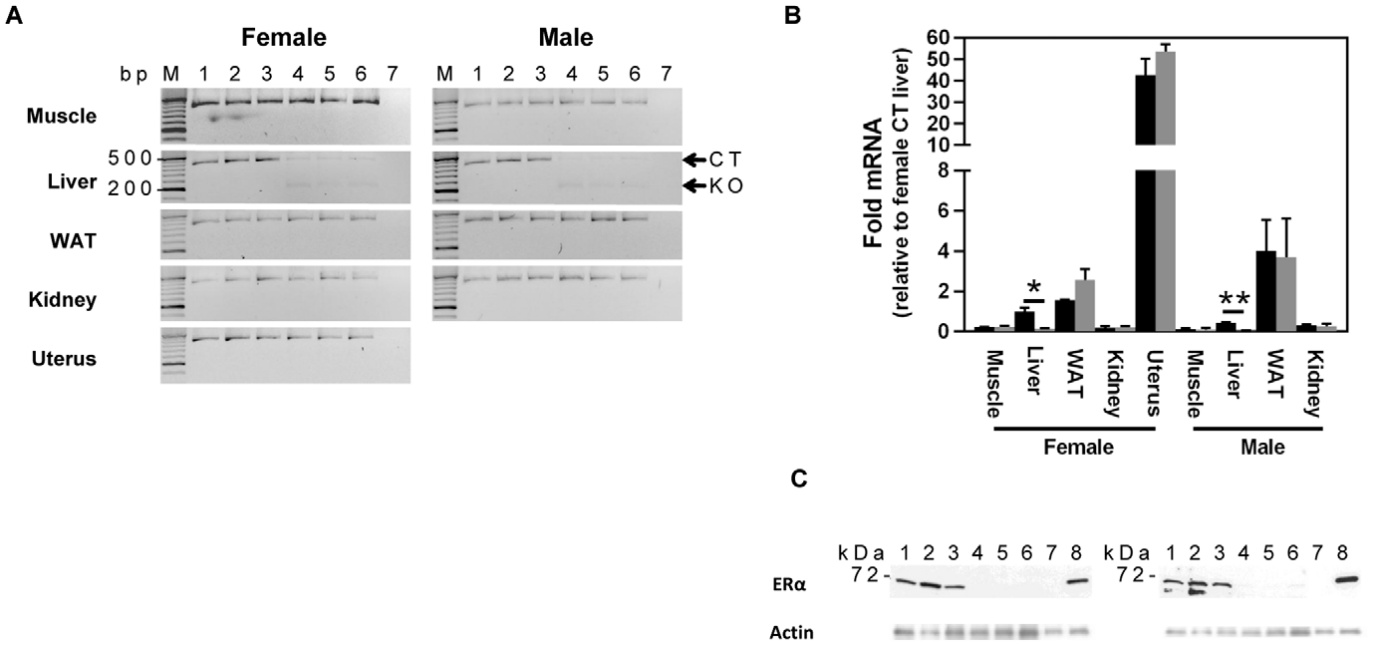
LERKO mice exhibit liver-specific downregulation of ERα. A) Downregulation of the ERα transcript is confined to the liver. Real-time PCR screening of ERα transcript across various LERKO tissues revealed the ERα transcript was significantly downregulated in the liver, but not in the muscle, white adipose tissue (WAT), kidney and uterus (female). B) Hepatic ERα transcript levels were approximately 10 fold lower in LERKO (grey bars) as compared to CT (black bars). Data are represented as mean ±SEM of three individual mice. * = P≤0.05; ** = P≤0.01. C) Western blot analysis of CT and LERKO liver lysates confirmed a strong downregulation of the ERα protein (but not actin) in LERKO livers. Uterus samples of wild type and ERαKO mice served as positive and negative controls, respectively. Actin was used as loading control. Each lane represents a single animal sample. Lanes 1–3 = WT; 4–6 = LERKO; 7 = ERαKO uterus; 8 = CT uterus. It is notable that a second band is detected by the ERα antibody in the liver of male but not female mice. While it is difficult to identify the exact source of the second band, one possibility is that it represents male prominent ERα degradation products. In line with this, longer exposure reveals a double band also in one of the liver samples from female mice (data not shown).

**Figure 2:**
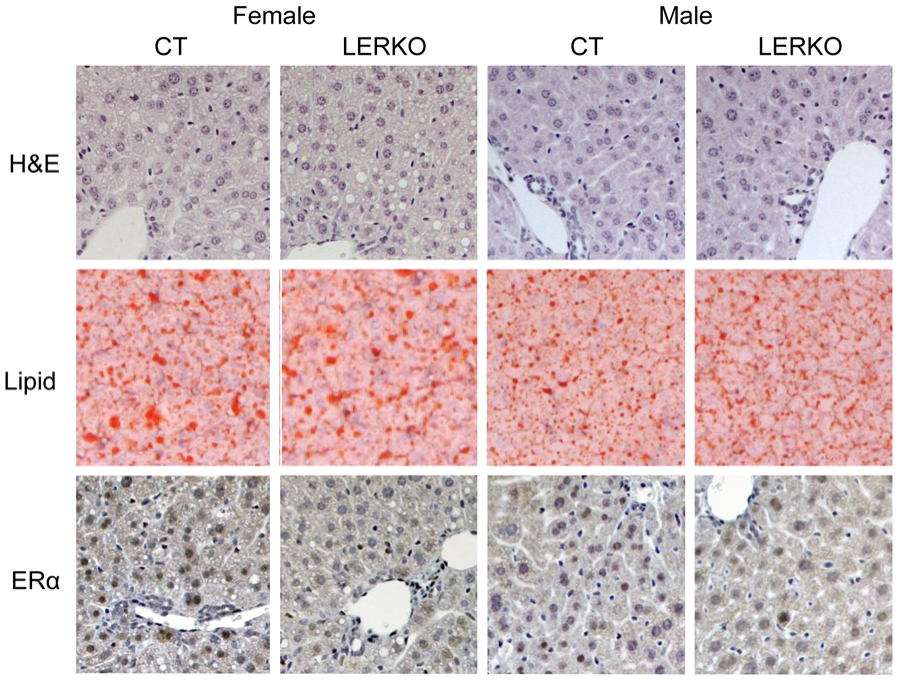
Immunohistochemical analysis of LERKO livers. Male and female liver tissue sections were analysed for gross structural morphology, lipid content and ERα protein expression. Hematoxylin and eosin (H&E) staining indicated CT and KO animals had similar gross liver morphology. Lipid staining revealed that similar amounts of lipid droplets were present in CT and LERKO animals. Staining for ERα indicated a predominant hepatic nuclear localisation, which was decreased in LERKO mice of both sexes. Sections from three individual 6 month-old mice from each of the test groups were analysed. The figure shows representative sections.

Successful liver-selective down-regulation of ERα was confirmed by evaluating the mRNA levels of ERα in muscle, liver, white adipose tissue (WAT), kidney, and uterus of LERKO and CT mice (Figure 1A). Significant down-regulation (of approximately 90%) of ERα mRNA levels was observed exclusively in the LERKO livers (Figures 1 A and B). Livers from CT and LERKO mice were further assessed for levels of ERα protein (Figure 1 C). Uterus samples of control (CT) and ERαKO mice served as positive and negative controls, respectively. As expected, the liver and uterus of CT, but not the uterus of ERαKO animals showed the presence of a ∼67 kDa ERα protein band. Densitometric analysis revealed that the protein band corresponding to ERα in male and female LERKO livers was <1% that of controls (data not shown). To ensure that the observed differences were not due to varying total protein levels, we confirmed that the total protein levels across all samples were approximately equal (Figure S1). Furthermore, actin levels were similar between LERKO and CT mice (Figure 1C). ERα protein levels were also assessed by immunostaining, which indicated that ERα was predominantly localised in the hepatocyte nucleus, and was of weaker intensity in LERKO compared to CT animals (Figure 2).

### LERKO animals have normal liver histology, body weight, glucose and insulin response

**Figure 3:**
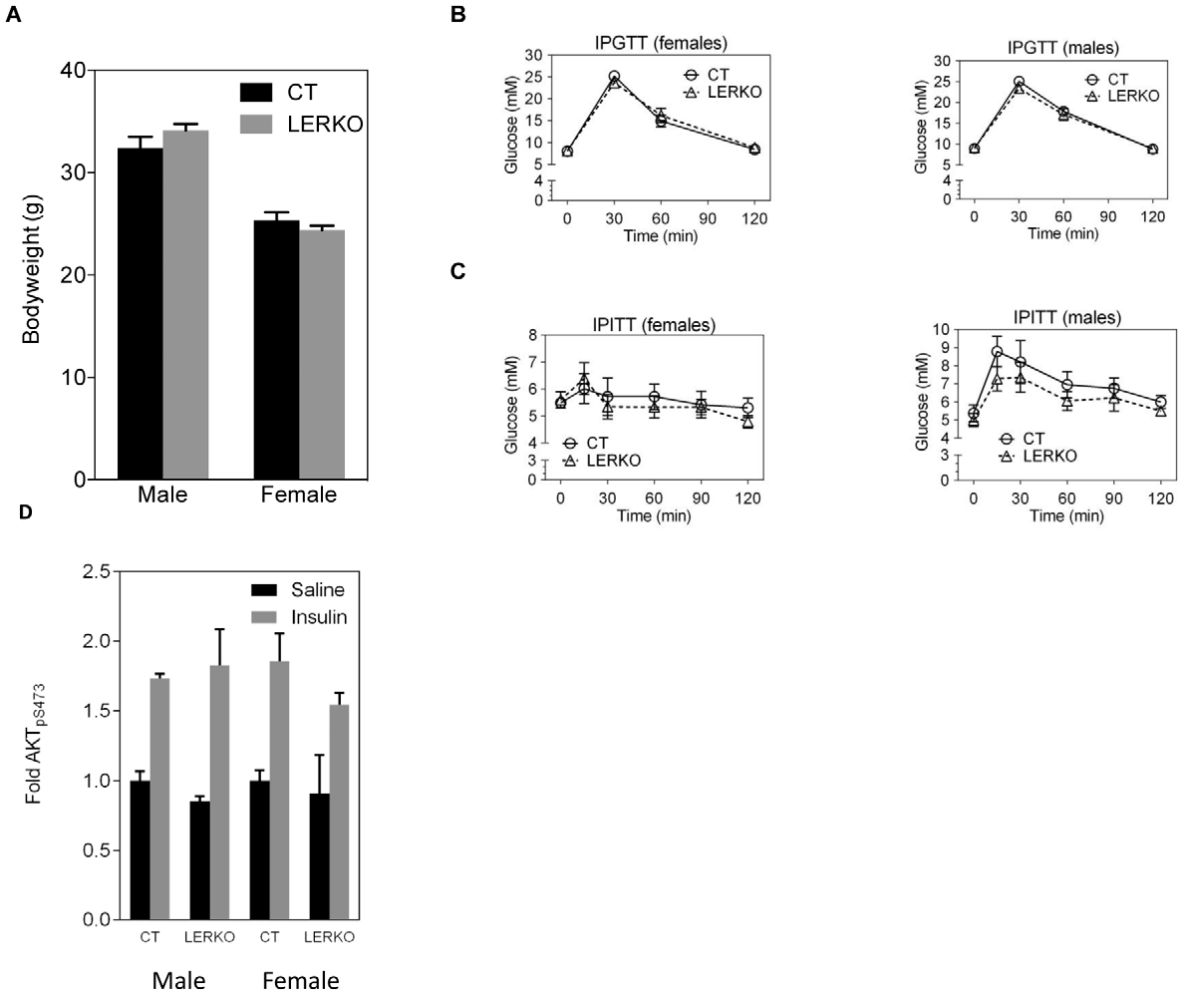
LERKO mice do not exhibit changes in body weight, glucose tolerance and insulin sensitivity. Body weight of 6 month old male and female LERKO mice was comparable to respective CT mice (A). LERKO and CT mice showed a similar glucose response when assessed by IPGTT (B) or IPITT (C). IPGTT data are represented as a mean ±SD. IPITT data are represented as a mean ±SEM. IPGTT males CT and LERKO n = 8, females CT n = 6, LERKO n = 8; IPITT males CT and LERKO n = 4, females CT and LERKO n = 7. For the IPGTTs and IPITTs we performed additional analyses, calculating the area under the curve. Using this analysis, there was no difference in insulin sensitivity between LERKO and CT mice for males and females (data not shown). Insulin-stimulated AKT phosphorilation in the liver was similar between CT and LERKO mice. Data are represented as a mean±SD. Males CT and LERKO n = 3, females CT and LERKO n = 2–4 (D).

**Figure 4:**
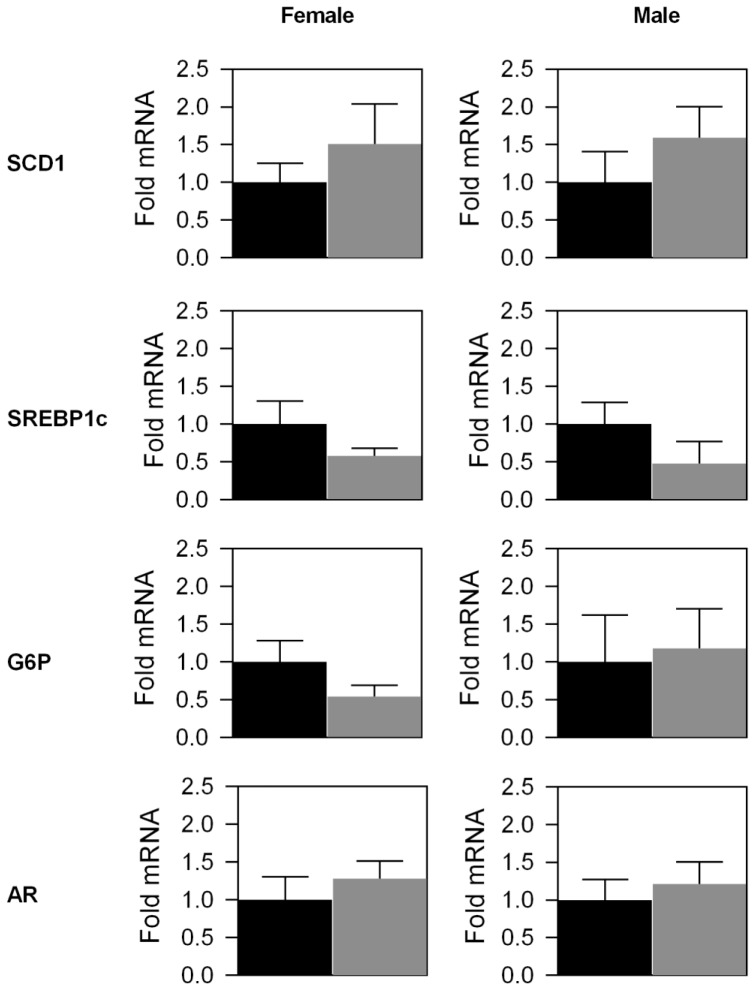
Quantitative PCR analysis of selected hepatic LERKO transcripts. Selected LERKO and CT liver transcripts were quantitatively analysed using qPCR. Significant changes in transcript levels were not observed for any of the evaluated transcripts. Data are represented as a mean ±SEM of three individual mice. Black bars = CT mice, grey bars = LERKO mice.

Analysis of gross liver tissue structure and lipid content in 6 month-old male and female LERKO and CT mice revealed no observable differences between LERKO mice and their respective controls (Figure 2 and Figure S2). As expected, we observed increased levels of lipid droplets in female mouse livers, compared to males [32]. Additionally, 6 month old LERKO and CT mice had comparable body weight, glucose tolerance and insulin sensitivity (Figures 3 A–C). Furthermore, insulin-stimulated AKT phosphorylation in the liver was similar between CT and LERKO mice (Figure 3 D). We also examined hepatic transcript levels of SREBP-1c, a classic indicator of hepatic steatosis [33], [34], [35]. We observed no differences in SREBP-1c levels between CT and LERKO mice of respective genders (Figure 4).

### The global hepatic gene expression profile from LERKO mice does not reflect the profile observed in ERαKO mice

In our previous study, we showed that livers from ERαKO mice demonstrated significant changes in the gene expression profile compared to CT mice [12]. To evaluate whether the hepatic transcriptional profile of LERKO animals shows similar changes to the previous observations in ERαKO animals, the LERKO hepatic gene expression was profiled by microarray expression analysis. Subsequently, the number of significantly regulated genes was evaluated and compared to the number of significantly regulated genes identified in ERαKO. Using a false discovery rate of 5%, we identified 3 significantly regulated genes (Table S1) in LERKO compared to 173 significantly regulated genes in ERαKO (Table S2). Of the 3 significantly regulated genes in LERKO mice, only the *Esr1* (coding for ERα) gene was also significantly regulated in ERαKO mice. Furthermore, we specifically evaluated hepatic mRNA expression levels of glucose-6-phosphatase (G6P), stearoyl-coenzyme A desaturase 1 (Scd1), ERβ, GPR30 and the androgen receptor (AR) (Figure 4; ERβ and GPR30 data not shown). We have previously shown that hepatic Scd1 and G6P expression levels are significantly affected by estrogenic signalling. In ERαKO mice, the hepatic Scd1 transcript is upregulated by ∼5 fold [12], while in HFD mice, E2 treatment decreased the expression levels of both G6P and Scd1 within the liver [20]. In addition, a decrease in hepatic G6P mRNA levels is also observed in ob/ob mice treated with E2 or PPT [4]. Thus, we proposed that hepatic Scd1 and G6P are likely to be mediators of the observed effects of estrogen signalling on metabolic phenotypes. However, in LERKO livers, Scd1 and G6P did not demonstrate any significant change in mRNA expression levels compared to CT livers (Figure 4).

Since both ERβ and GPR30 can mediate estrogen signalling [27], [28], [36], we speculated that these signalling pathways could compensate for the reduced hepatic ERα signalling. However, in CT and LERKO mice, hepatic mRNA levels of ERβ were undetectable while GPR30 mRNA levels were very low and of a similar level (data not shown) suggesting that ERβ and GPR30 signalling did not have a compensatory role in the livers of LERKO mice. Additionally, AR signalling within the liver has recently been implicated in hepatic glucose and lipid homeostasis [37]. We measured the hepatic AR transcript to evaluate whether increased hepatic AR levels could be involved in the maintenance of insulin sensitivity in LERKO mice. However, LERKO and CT mice had comparable hepatic AR transcript levels (Figure 4).

### Body weight, glucose response and hormone levels of LERKO mice are comparable to CT mice when challenged with a high fat diet and/or age

**Figure 5:**
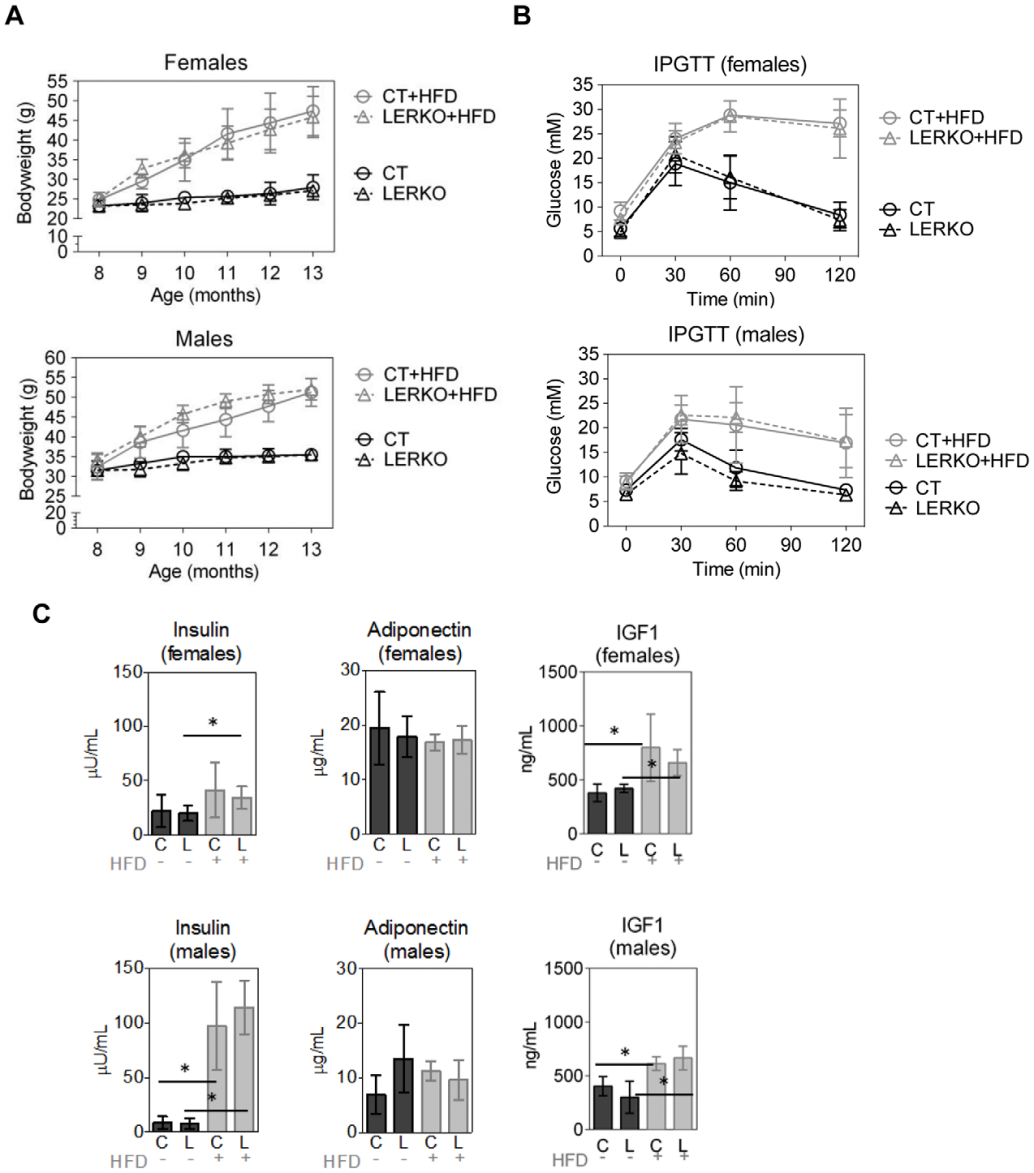
HFD-fed LERKO mice show normal hormone profiles and insulin and glucose responses. Eight month-old LERKO and CT mice were fed with a high fat or standard chow diets for 5 months, and changes in body weight (A), glucose tolerance (B) and circulating hormones (C) were assessed. Body weight increased steadily in HFD animals across both sexes. However, the increase in body weights was comparable in the CT and LERKO mouse groups within the respective genders (A). Male chow diet CT/LERKO n = 4; HFD CT/LERKO n = 8. Female chow CT/LERKO n = 7; HFD CT n = 6, LERKO n = 8. (B) IPGTT assessment of standard chow-fed and HFD regimented groups revealed that the HFD groups exhibited pronounced impaired glucose tolerance. However, comparable responses during IPGTT were observed between the respective male or female CT and LERKO mouse groups. Male chow CT/LERKO n = 4; HFD CT n = 7, LERKO n = 8. Female chow CT/LERKO n = 7; HFD CT n = 5, LERKO n = 7. (C) Compared to standard chow-diet, HFD feeding lead to increased insulin and IGF-1 levels both in CT and LERKO mice. However, circulating hormone levels were similar in CT and LERKO mice maintained on HFD or standard chow diet. n = 4–8. Additionally, significant difference was not observed for IPGTT based on calculating the area under the curve (data not shown).All data are presented as mean ±SD.

To challenge the LERKO model, 8 month-old LERKO and CT mice were subjected to a 5 month HFD. As expected, the male and female HFD regimented mice showed a marked increase in body weight over the course of the diet, compared to age-matched standard chow-fed male and female mice (Figure 5 A). Importantly, there were no significant differences in body weights between male or female CT and LERKO mice (Figure 5 A). HFD-fed mice displayed pronounced reductions in glucose tolerance. However, no differences in glucose tolerance were observed between the CT and LERKO mice (Figure 5 B). CT and LERKO mice receiving the standard diet showed normal glucose tolerance (Figure 5 B). We determined levels of insulin and adiponectin as markers of insulin resistance, and of IGF-1 as a hormone sensitive to the diet. Analysis of circulating hormone levels revealed that CT and LERKO mice had comparable insulin, IGF-1 and adiponectin levels, within their respective sexes and for both diets (Figure 5 C). HFD-fed mice showed increased levels of insulin and IGF-1, while adiponectin levels were comparable for chow- and HFD-fed mice.

## Discussion

We previously demonstrated that ERαKO mice have pronounced hepatic insulin resistance [12]. To determine whether liver-selective ablation of ERα recapitulates the metabolic phenotypes of ERKO mice, we generated a liver-selective ERαKO mouse model, LERKO. The resulting LERKO mice displayed an efficient ablation of ERα expression selectively within the liver (Figures 1 and 2), confirming the successful generation of a liver tissue selective ERα KO model. We have previously shown that ERαKO mice display an increase in body weight, impaired glucose tolerance and insulin resistance [12]. In contrast, compared to CT mice, LERKO mice maintained comparable body weights, and responded similarly during glucose and insulin tolerance tests compared to CT mice even when challenged with a HFD or age (Figures 3 and 5). There were no differences either in the basal or glucose-stimulated insulin responses during the GTT between CT and LERKO mice (data not shown). Thus LERKO mice do not secrete more insulin to maintain a glucose response similar to that of CT mice during the GTT. Furthermore, to make sure that we did not miss an early and transient phenotype, we also measured glucose tolerance at 3 months of age and observed similar glucose tolerance for LERKO and CT mice for males and females (data not shown). Additionally, a key mediator of insulin signalling, phosphorylation of Akt, was similar between CT and LERKO mice for the liver. Thus LERKO mice maintain normal liver insulin sensitivity.

Della Torre S et al. [38] showed a small decrease in circulating levels of IGF-1 between CT and LERKO mice under specific dietary conditions. We do not observe any difference in circulating IGF-1 levels between CT and LERKO mice on a standard chow diet or on a HFD. However feeding with HFD resulted in increased level of IGF-1 both in CT and LERKO mice.

Together, these observations indicate that selectively ablating ERα action in the liver is not sufficient to recapitulate the metabolic phenotype observed in mice with whole body disruption of ERα. Our western blot analyses indicate that the remaining ERα in the liver of LERKO mice corresponds to less than 1% of that in livers from CT mice (Figure 1). The most likely source of the remaining ERα are the non-parenchymal cell types, as these have been noted not to express the albumin promoter used to drive the cre expression in LERKO mice [32], [33]. Therefore, it remains possible that ERα signalling in non-parenchymal cells might have an important role in the ERα-mediated hepatic insulin resistance observed in ERαKO mice. In support of this possibility, a recent study showed that Kupffer cells (a hepatic non-parenchymal cell type) mediated responses that contribute to the onset of HFD-induced hepatic insulin resistance [39]. However, whether Kupffer cells contribute to hepatic insulin resistance in the absence of HFD, as observed in ERαKO mice and whether the observed Kupffer cell mediated effect is dependent on ERα-mediated signalling remains to be elucidated.

Other studies have noted that the albumin promoter is not fully active until the mice are 6 weeks of age [40]. Since ERαKO mice are ERα-null during their entire development, it is possible that the onset of the observed phenotype in ERαKO mice is dependent on ERα signalling during an early developmental phase.

Another possible explanation for the absence of an observable metabolic phenotype in LERKO mice could be the presence of compensatory mechanisms. Previous studies utilising liver-selective AR KO mice have implicated AR as a positive factor in preventing the development of hepatic steatosis and insulin resistance [37]. In addition, studies in mice lacking G protein-coupled receptor (GPR) 30, a functional estrogen receptor, have established GPR30 as an important factor in insulin sensitivity and glucose homeostasis [28]. Furthermore, since ERβ is known to respond to estrogens [41], and associate with ERα related gene targets [41], it is possible that ERβ could supplement the ERα function when ERα is low. All three of these proteins are potential candidates in driving potential compensatory mechanisms. However, we did not find any significant differences in AR, GPR30 or ERβ transcript levels between livers of LERKO and CT mice, suggesting that these proteins are not involved in compensatory mechanisms in LERKO livers.

Our previous study of ERαKO mice demonstrated a moderate decrease in insulin stimulated glucose uptake in the muscle *in vitro,* suggesting that the muscle contributes to the metabolic phenotypes observed in ERαKO mice. This is consistent with the data reported by Ribas et al. [42], which shows a contribution by muscle to glucose disposal *in vivo.* Further studies using mice with skeletal muscle-selective ablation of ERα will further shed light on the contribution of ERα in the muscle to the observed phenotypes in ERαKO mice.

While an unknown compensatory mechanism might still be responsible for maintaining normal body weight, and glucose homeostasis in the LERKO mouse model, it is important to note that our gene expression profiling analysis indicated only 3 genes out of the estimated ∼28000 genes detectable by the utilised microarray had altered transcript levels between LERKO and CT mice (Table S1). Importantly, the presence of *Esr1* in this category confirmed the successful downregulation of the ERα transcript in the LERKO mouse model. The remaining 2 genes were not identified as significantly changed in the corresponding ERαKO analyses (Table S2), thus further investigation is needed to evaluate the relevance of ERα-mediated signalling for the regulation of these genes. We speculate that the absence of further differences between the LERKO and CT hepatic transcriptional profiles does not support the presence of compensatory actions, which are likely to be reliant on alteration of metabolic pathways to maintain homestatic balance. However, it still remains possible that compensatory mechanisms working through metabolic pathways identical to ERα could still be present.

The MS is a complex multifactorial syndrome, involving multiple organs [43]. The exact sequence of events and the role of estrogenic action in the development of the MS remain to be elucidated. While our results suggest that downregulation of hepatic ERα action does not induce the development of insulin resistance and obesity, the possibility remains that ERα signalling has crucial roles downstream of the initiating factors.

In this study, we pursued a logical continuation of our previous work in delimitating the functional contribution of hepatic ERα for the previously observed hepatic insulin resistance exhibited by total ERαKO mice. However, our results indicate that downregulating ERα in the liver does not recapitulate the previously observed ERαKO phenotype. Whether this lack of an ERαKO-like phenotype in LERKO mice could be due to unidentified compensatory mechanism/s, or whether hepatic insulin resistance occurs as a secondary/downstream effect upon ablation of estrogen signalling in other cell types, remains to be elucidated.

## Materials and Methods

### Ethics statement

The ‘Stockolms Södra and Norra Djuretiska Nämnd’ ethics committees approved all animal experiments (approval numbers: S10/09, S11/09 and N398/10).

### Animals

C57BL/6J mice expressing the cre recombinase under the liver specific albumin promoter (B6.Cg-Tg(Alb-cre)21Mgn/J) were purchased from The Jackson Laboratory. The generation of floxed ERα mice has been described elsewhere [44]. To generate mice with a liver-specific knockout of ERα, the Alb-cre transgenic mice were crossed with ERα^fl/fl^ animals to obtain Alb-cre/ERα^fl/+^. These mice were subsequently crossed with ERα^fl/fl^ animals, resulting in Alb-cre/ERα^fl/fl^ mice (LERKO). ERα^fl/fl^ animals served as controls. Genotyping for the Alb-cre transgene was performed with the following primers: F: 5′-TAATGGGGTAGGAACCAATG-3', R: 5'-GTTTCACTATCCAGGTTACGG-3'. Genotyping of the floxed-ERα locus was performed as described elsewhere [44]. Male and female LERKO mice were fertile with the females exhibiting a regular estrous cycle (data not shown). All animals were maintained on 12 h light-dark cycle, with food and water available *ad libitum.* From 8 to 13 months of age male and female LERKO mice, together with age-matched CT mice, were maintained on chow diet or on a HFD containing 34.9 g% fat, 26.2 g% protein and 26.3 g% carbohydrate (Research Diet, New Brunswick, NJ, USA). At the end of the experiment the mice were decapitated, blood was collected in heparinized tubes, centrifuged, and plasma was stored at −20C. Liver, adipose tissue, muscle, kidney and uterus were removed and stored at −80C.

### *Circulating hormone analyses*

Plasma insulin levels were measured by RIA using ^125^I-labeled porcine insulin, guinea pig anti-porcine serum and rat insulin as a standard (Novo Nordisk, Denmark). Plasma adiponectin levels were assessed with a double-antibody RIA technique in which ^125^I-labeled murine adiponectin, multispecies adiponectin rabbit antiserum and mouse adiponectin standard were used (Millipore, Billerica, MA, USA). Plasma levels of IGF-1 were analysed by mouse/rat IGF-1 ELISA (Mediagnost, Germany).

### *Intraperitoneal glucose tolerance test (IPGTT)*

In overnight-fasted mice, blood glucose concentrations were measured before and after (30, 60 and 120 min) the intraperitoneal injection of glucose at a dose of 2 g/kg. Blood glucose concentrations were analysed using the MediSence glucose analyser (Abbott Scandinavia AB, Solna, Sweden).

### *Intraperitoneal insulin tolerance test (IPIIT)*

IPITT was performed in overnight fasted mice. Blood glucose concentrations were initially measured at the basal condition (0 min), then the animals were administered an intraperitoneal injection of insulin (0.25 U/kg) (Actrapid, Novo Nordisk) followed by an intraperitoneal injection of glucose (1 g/kg). Subsequently, blood glucose concentrations were measured at 15, 30, 60, 90 and 120 min after the glucose load.

### Insulin signalling in vivo

Overnight-fasted animals were injected intraperitoneally with saline or human insulin (Actrapid, Novo Nordisk) at a dose 2 U/kg. After 5 min, mice were sacrificed, tissues were harvested and stored at −80°C.

### Assessment of AKT[pS473]

Liver protein extracts were prepared by homogenizing tissue in RIPA buffer, followed by centrifugations at 10 000×g for 10 min. Supernatants were transferred to new tubes and centrifuged again as previously. Finally, the supernatants were centrifuged at 14 000×g for 10 min. Protein concentrations of the extracts were determined using the BCA Protein Assay (Thermo Scientific, USA). Akt[pS473] was assessed by ELISA (BioSource, Belgium).

### Immunohistochemistry

Paraffin-embedded tissue blocks were cut at 4μm thickness, de-paraffinized, and rehydrated. Antigen retrieval was executed by microwaving the sections at 650 W in 10 mM citrate buffer (pH 7.0) for 15 min. Endogenous tissue peroxidase was then quenched by immersion in 0.5% H_2_O_2_/PBS for 30 min/RT, then 0.5% Triton X-100/PBS for 15 min. To minimise non-specific antibody binding, sections were treated with BlockAce (Dai-nippon Pharmaceutical, Japan) for 40 min/RT. The anti-ERα (Santa Cruz, MC-20: sc-542) primary antibody (1∶250 dilution) was applied to the sections overnight/4°C in 10% BlockAce/PBS. Subsequently, the sections were washed and incubated for 1 h/RT with appropriate biotinylated secondary antibody (1∶200 dilution). Visual staining was achieved with the avidin-biotin complex (ABC) method [45] with the Vectastain ABC kit (Vector). Peroxidase activity was visualized with 3,3'-diaminobenzidine (DAKO). Sections were lightly counterstained with hematoxylin. Negative controls were treated equally, without incubation with primary antibodies.

### Oil Red O Staining

Fresh liver tissue were immersed in Tissue-Tek OCT compound (Sakura, Japan) and then frozen in isopentane cooled by liquid nitrogen. Samples were subsequently stored at -80°C. Stock Oil Red O solution was made by dissolving 300 mg of Oil red O powder (Sigma) in 100 mL of 99% isopropanol. Mixing, then filtering, 60 ml of the stock solution with 40 ml of distilled water produced the working solution, which was used within 1 h. Twelve micrometer frozen liver cryosections were air dried, incubated in Oil Red O working solution for 30 min then washed in distilled water. Sections were subsequently lightly counterstained with hematoxylin.

### RNA extraction

Total RNA was isolated from frozen mouse tissues using the Trizol reagent (Invitrogen) as per the manufacturer's instructions. Isolated RNA was subsequently, purified with the RNeasy Plus Mini Kits (Qiagen), and quantitated using a NanoDrop 1000 spectrophotometer (Thermo Scientific) and the accompanying software.

### Microarray Analysis

All microarray experiments have been performed in accordance to the MIAME microarray experiment guidelines [32]. The gene expression profile of liver in ERαKO mice was determined using Affymetrix MOE430 A arrays as reported previously [12]. In the current study, Affymetrix Mouse Gene 1.1 ST arrays were used for analysis of liver gene expression in LERKO mice. To facilitate the comparison between the ERαKO and LERKO data sets all microarray data were analysed or re-analysed with related packages available from Bioconductor [46]. Normalization and calculation of gene expression was performed with the Robust Multichip Average (RMA) expression measure using the affy package and oligo packages respectively for the ERαKO and LERKO studies. Prior to further analysis, a nonspecific filter was applied to remove genes with expression signals below 100 across all samples. Significant differential expression between KO and CT groups was assessed with the limma package, mean fold changes were estimated, and a false discovery rate of 5% was employed. The data discussed in this publication have been deposited in NCBI's Gene Expression Omnibus [47] and are accessible through GEO Series accession number GSE36514 (http://www.ncbi.nlm.nih.gov/geo/query/acc.cgi?acc= GSE36514).

### PCR and agarose gel electrophoresis

PCR analysis was performed using BIO-X-ACT Short Mix (Bioline) according to the manufacturer's instructions. One microliter of cDNA was used as the starting template together with the following primers flanking exon 3 of *mERα*: F:5′-CACGGCCAGCAGGTGCCCTA-3′, R: 5′-GGCCTGGCAACTCTTCCTCCG-3′. The applied thermocycling regime employed was: 94°C, 2 min; [94°C, 30 s; 60°C, 30 s; 72°C, 30 s] for 30 cycles; 72°C, 5 min. The reaction products were resolved on 3% agarose gels (UltraPure Agarose 1000, Sigma) in TBE buffered conditions.

### Quantitative real-time PCR

Individual cDNA samples were assessed for gene expression by quantitative real-time PCR using the 7500 Fast Real-Time PCR System (Applied Biosystems) with the Fast SYBR Green master mix (Applied Biosystems) according to the accompanying protocol. Melting curve analyses was applied to confirm system/primer specificity. Relative gene expression was evaluated using either the Standard Curve (Applied Biosystems, User Bulletin #2) or, where appropriate, the comparative CT method [48]. Gene expression was normalised to murine glyceraldehyde-3-phosphate dehydrogenase (mGAPDH). Targeted qPCR primers utilised were *mERβ* (F:GCCAACCTCCTGATGCTTCT; R:TCGTACACCGGGACCACAT), *mERα* (F:GAGAAGCATTCAAGGACACAATGA; R: CGGTTCTTGTCAATGGTGCAT), *mGAPDH*: (F:TGTGTCCGTCGTGGA; R:CCTGCTTCACCACCT), *mCyp3a41*: (F:GTGGAGAAAGCCAAAGGGATT, R:GAAGACCAAAGGATCAAAAAAGTCA), *mScd1*: (F:CCGGAGACCCCTTAGATCGA, R:TAGCCTGTAAAAGATTTCTGCAAA), *mG6P*: (F:TCCGTGCCTATAATAAAGCAGTT, R:GTAGAAGTGACCATAACATAGTA), *mFmo3*: (F:CAGCATTTACCAATCGGTCTTC, R: TGACTTCCCATTTGCCAGTAG), *mHsd3b5*: (F:GCCTGGAACCTCTTGTAGGTAGAA, R:GAAATGCTTTGGCACATGGA), *mGPR30*: (F:GACTCTGCTCCCCTTAAGCTG, R:GAAAGATAAACCAGGCATTTG), *mAR*: (F:TGCTCTACTTTGCACCTGACTTG, R:ACTGGCTGTACATCCGAGACTTG), *mIGF1*: (F:TGCCCAGCGCCACACT, R: TTCGTTTTCTTGTTTGTCGATAGG), *mSREBP-1c*: (F: GGAGCCATGGATTGCACATT, R: GCTTCCAGAGAGGAGGCCAG). All primers are shown in the 5′ to 3′ orientation.

### Western blot analysis

Frozen tissue was homogenized in RIPA Buffer (Sigma) containing complete mini protease inhibitor cocktail (Roche). To ensure equal loading, protein concentrations were determined using the BCA Protein Assay Kit (Pierce). Protein extracts (100μg liver, 5μg uterus) were resolved on a 7.5% Mini-PROTEAN TGX Precast Gel (Biorad) then transferred to Amersham Hybond-LFP membranes (GE Healthcare). Membranes were blocked with 5% skimmed milk (1 h, RT), then incubated overnight at 4°C with the anti-ERα antibody [1∶200 dilution] (Santa Cruz, MC-20: sc-542) or anti-Actin antibody [1∶10000] (Sigma Aldrich) under gentle agitation. Subsequently, the membranes were washed (3×15 min) in PBS containing 1% Tween-20 (Duchefa Biochemie), then incubated with the anti-rabbit [1∶2000 dilution] (for ERα) or anti-mouse [1∶5000 dilution](for Actin) secondary antibody for 1 h at room temperature under gentle agitation. Following a repetition of the washing steps, the antibody targeted proteins were visualized with the use of the Pierce SuperSignal West Pico Chemiluminescent Substrate kit (Thermo Scientific) followed by autoradiograph film exposure. To ensure the observed differences in band intensity were not due to differential protein concentration, membranes were checked for equal lane loading by Coomassie R-350 staining as previously described [49]. Densitometric analysis was performed with ImageJ [50], utilising coomassie staining for normalisation of ERα band intensity.

### Statistical analysis

Unless otherwise stated, quantitative data are expressed as mean ±SD. Statistical significance was assessed using the two-tailed Student's t-test assuming unequal variance. Figures 5A and 5 C were analyzed using two-way repeated measurements ANOVA or two way ANOVA, respectively, followed by the Tukey post-hoc test. Significance was established at P≤0.05 and represented as an asterix (*).

### Analysis of hepatic lipid composition

Hepatic lipids were extracted as previously described [51], [52]. In brief, ∼100 mg of liver was extracted in chloroform-methanol (2∶1, v/v), solubilised in 1% Trition X-100 solution, and total cholesterol and triglycerides were determined by enzymatic assays. Cholesterol and triglyceride reagents were purchased from Roche Diagnostics (GmbH, Mannheim, Germany).

## Supporting Information

Figure S1 **Confirmation of equal protein loading in ERα targeted Western Blot analysis.** Coomassie staining of membranes probed for ERα protein (Figure 1C) revealed similar total protein levels across CT and LERKO sample lanes. Lanes M = marker; 1–3 = CT; 4–6 = LERKO; 7 = ERKO uterus; 8 = CT uterus.+
(TIF)

Figure S2 **Control and LERKO mice exhibit similar levels of hepatic lipid content within respective gender.** Male CT n = 3, LERKO = 3. Female CT = 3, LERKO = 3. Data are presented as mean ±SD.
(TIF)

Table S1 **Significantly changed genes in CT versus LERKO mice.** Genes identified as having a significant change in the hepatic transcriptional levels between LERKO and CT mice. FDR = false discovery rate.
(XLSX)

Table S2 **Significantly changed genes in CT versus ERαKO mice.** Genes identified as having a significant change in the hepatic transcriptional levels between ERαKO and CT mice. FDR = false discovery rate.
(XLSX)
