# Correction: Estrogen Signalling and the Metabolic Syndrome: Targeting the Hepatic Estrogen Receptor Alpha Action

**DOI:** 10.1371/journal.pone.0217526

**Published:** 2019-06-19

**Authors:** Marko Matic, Galyna Bryzgalova, Hui Gao, Per Antonson, Patricia Humire, Yoko Omoto, Neil Portwood, Camilla Pramfalk, Suad Efendic, Per-Olof Berggren, Jan-Åke Gustafsson, Karin Dahlman-Wright

There is an error in Fig 2. The wrong image was included as part of the final revision process such that lipid data for female controls were duplicated and shown as also representing female LERKO. The authors have provided the correct version here after a re-analysis of the original samples to confirm the results presented in Fig 2, as the original raw image underlying Fig 2 is no longer available. Raw data for all figures (including the data for the supplementary figures and tables) are available except for:

- Fig 1C: The panel for Western blot analysis of ERα in male liver lysate.
- Fig 2: All the raw data for this figure as stated above.
- S1 Fig: Coomassie-blue staining of membranes for male samples.

The original female liver samples from the study have been re-analyzed for liver lipids. The conclusion that analysis of lipid content revealed no observable differences between female LERKO and control mice is confirmed. Representative images are shown in S1 File, Fig 2R. Quantification of images are shown in S1 File, Fig 2RB. This quantification was not performed in the published paper.

Liver samples from female LERKO and Control mice (3 animals in each group) were analyzed at the core facility of morphological phenotype analysis (FENO), Karolinska Institutet, by Oil-Red-O staining. Briefly, frozen liver tissues were immersed in OCT Cryomount (Histolab, Sweden), then frozen and sectioning at six micrometer. Frozen liver cryo sections were air dried, incubated in Oil-Red-O working solution for 3 min then washed in distilled water. Sections were subsequently lightly counterstained with hematoxylin.

Image analysis of lipid content was performed with the Histoquant module in 3DHISTECH Panoramic Viewer. Five areas were selected from the slides for each mouse liver, and the percentage of the area covered with Oil-Red-O was calculated for each selected area. The Cumulated percentage of the areas covered with Oil-Red-O staining was then calculated for each mouse.

## Supporting information

S1 File Reanalysis of Fig 2. Liver lipids in LERKO and control female livers **A.** Female liver tissue sections were analyzed for lipid content. Lipid staining reveals similar amounts of lipid droplets in livers of control (CT) and LERKO animals. Representative images are shown. **B.** Percentage of areas with Oil-Red-O staining in the livers from female CT and LERKO mice (n = 3).
(TIF)
