# Supplementary figures and images for: Estrogen Signalling and the Metabolic Syndrome: Targeting the Hepatic Estrogen Receptor Alpha Action
Source: PLoS One. 2013 Feb 25;8(2):e57458. doi: 10.1371/journal.pone.0057458 (PMC3581463; doi:10.1371/journal.pone.0057458)

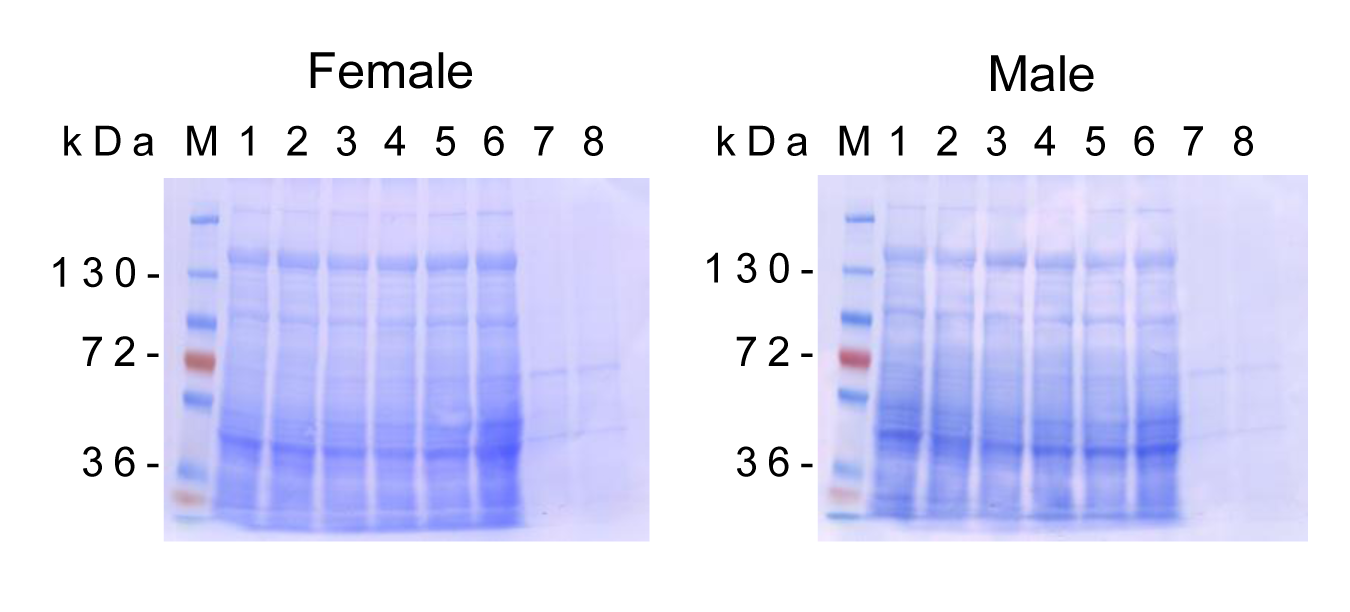

Supplement: Figure S1 — Confirmation of equal protein loading in ERα targeted Western Blot analysis. Coomassie staining of membranes probed for ERα protein (Figure 1C) revealed similar total protein levels across CT and LERKO sample lanes. Lanes M = marker; 1–3 = CT; 4–6 = LERKO; 7 = ERKO uterus; 8 = CT uterus.+ (TIF) [file pone.0057458.s001.tif]

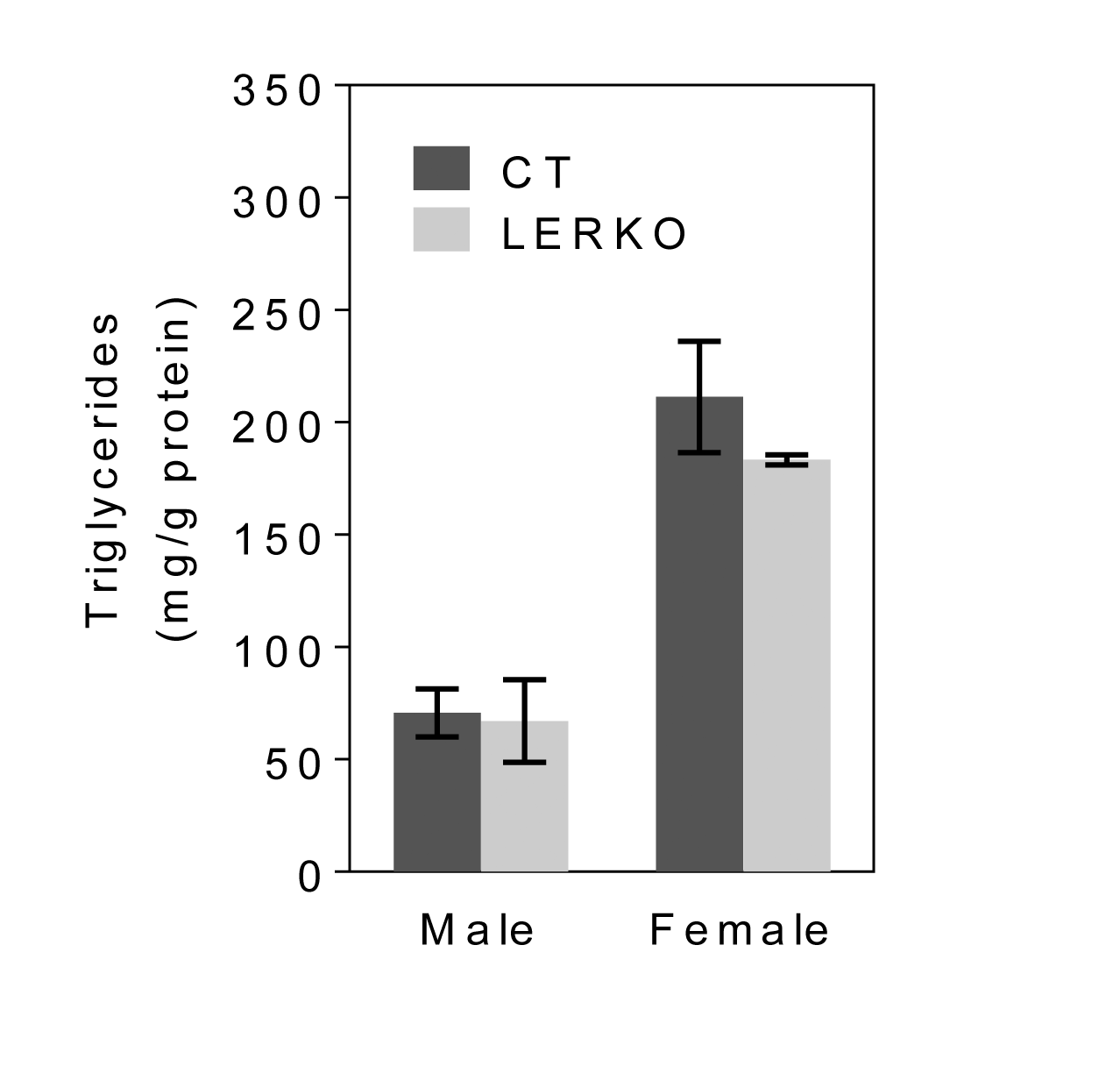

Supplement: Figure S2 — Control and LERKO mice exhibit similar levels of hepatic lipid content within respective gender. Male CT n = 3, LERKO = 3. Female CT = 3, LERKO = 3. Data are presented as mean ±SD. (TIF) [file pone.0057458.s002.tif]
