# Supplementary figures and images for: Correction: Estrogen Signalling and the Metabolic Syndrome: Targeting the Hepatic Estrogen Receptor Alpha Action
Source: PLoS One. 2019 Jun 19;14(6):e0217526. doi: 10.1371/journal.pone.0217526 (PMC6583955; doi:10.1371/journal.pone.0217526)

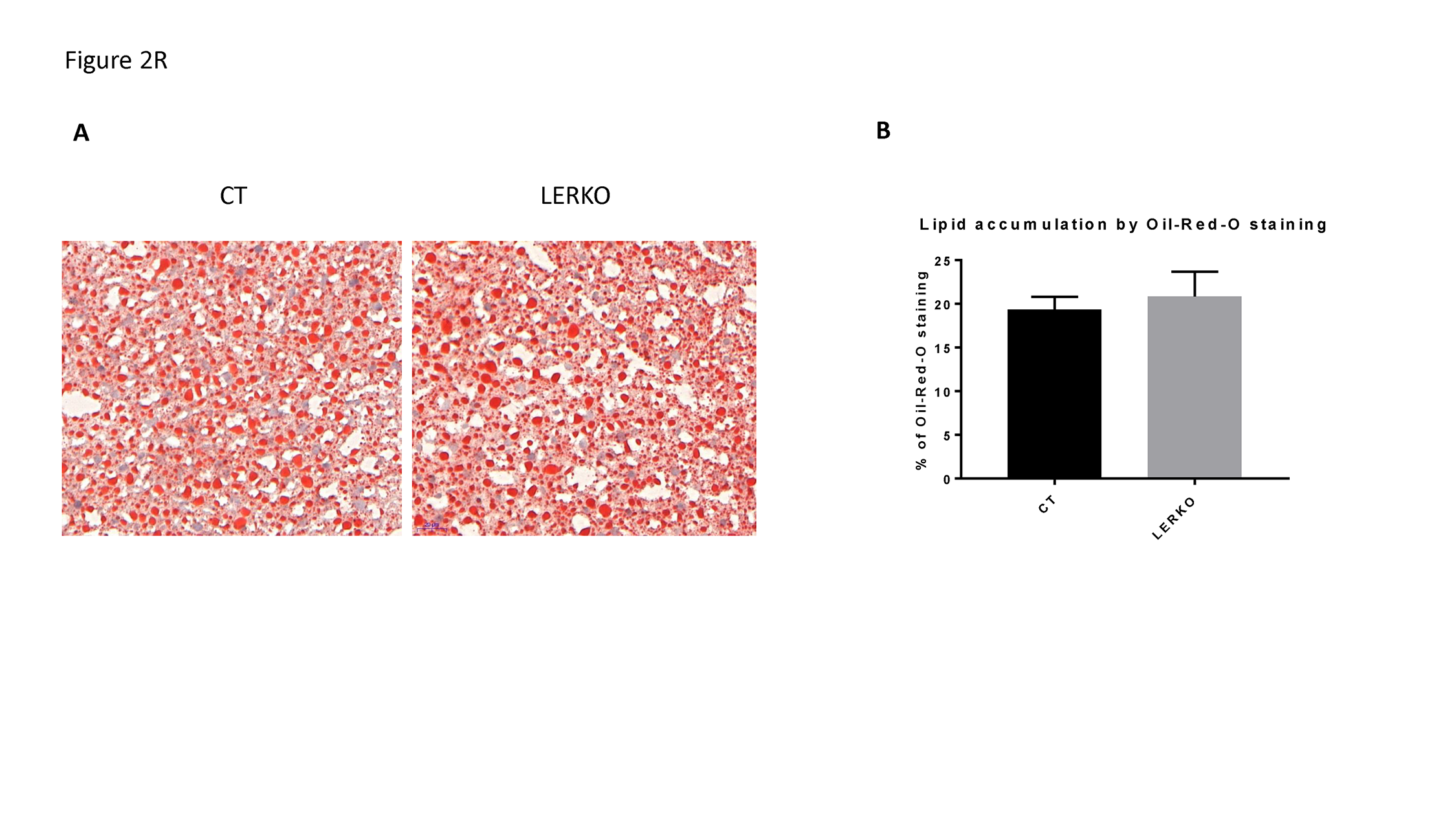

Supplement: S1 File — Liver lipids in LERKO and control female livers A. Female liver tissue sections were analyzed for lipid content. Lipid staining reveals similar amounts of lipid droplets in livers of control (CT) and LERKO animals. Representative images are shown. B. Percentage of areas with Oil-Red-O staining in the livers from female CT and LERKO mice (n = 3). (TIF) [file pone.0217526.s001.tif]
